# Endogenous IL-1 in Cognitive Function and Anxiety: A Study in IL-1RI^−/−^ Mice

**DOI:** 10.1371/journal.pone.0078385

**Published:** 2013-10-30

**Authors:** Carol L. Murray, Pauline Obiang, David Bannerman, Colm Cunningham

**Affiliations:** 1 School of Biochemistry and Immunology & Trinity College Institute of Neuroscience, Trinity College Dublin, Dublin, Republic of Ireland; 2 Department of Experimental Psychology, University of Oxford, Oxford, United Kingdom; Universidade de São Paulo, Brazil

## Abstract

Interleukin-1 (IL-1) is a key pro-inflammatory cytokine, produced predominantly by peripheral immune cells but also by glia and some neuronal populations within the brain. Its signalling is mediated via the binding of IL-1α or IL-1β to the interleukin-1 type one receptor (IL-1RI). IL-1 plays a key role in inflammation-induced sickness behaviour, resulting in depressed locomotor activity, decreased exploration, reduced food and water intake and acute cognitive deficits. Conversely, IL-1 has also been suggested to facilitate hippocampal-dependent learning and memory: IL-1RI^−/−^ mice have been reported to show deficits on tasks of visuospatial learning and memory. We sought to investigate whether there is a generalised hippocampal deficit in IL-1RI^−/−^ animals. Therefore, in the current study we compared wildtype (WT) mice to IL-1RI^−/−^ mice using a variety of hippocampal-dependent learning and memory tasks, as well as tests of anxiety and locomotor activity. We found no difference in performance of the IL-1RI^−/−^ mice compared to WT mice in a T-maze working memory task. In addition, the IL-1RI^−/−^ mice showed normal learning in various spatial reference memory tasks including the Y-maze and Morris mater maze, although there was a subtle deficit in choice behaviour in a spatial discrimination, beacon watermaze task. IL-1RI^−/−^ mice also showed normal memory for visuospatial context in the contextual fear conditioning paradigm. In the open field, IL-1RI^−/−^ mice showed a significant increase in distance travelled and rearing behaviour compared to the WT mice and in the elevated plus-maze spent more time in the open arms than did the WT animals. The data suggest that, contrary to prior studies, IL-1RI^−/−^ mice are not robustly impaired on hippocampal-dependent memory and learning but do display open field hyperactivity and decreased anxiety compared to WT mice. The results argue for a careful evaluation of the roles of endogenous IL-1 in hippocampal and limbic system function.

## Introduction

Interleukin 1 (IL-1) is a key pro inflammatory cytokine that is primarily produced in the periphery by immune cells but can also be synthesized by glia and neurons within the brain. Interleukin 1 signalling is mediated by the binding of IL-1 α or IL-1β to the interleukin type one receptor (IL-1R1). Interleukin 1 signalling can be blocked by IL-1 receptor antagonist (IL-1ra) which binds to the IL-1R and blocks signal transduction (see [1] for review). Roles of IL-1 have been investigated using mice deficient in the IL-1RI.

Interleukin 1 (IL-1) has been shown to play a key role in sickness behaviour resulting in depressed locomotor activity, decreased exploration, reduced food and water intake, [2]. Furthermore, significant data have accumulated to implicate IL-1 in cognitive impairments during acute inflammation. Most of these studies would appear to implicate acutely synthesised IL-1 in the disruption of memory consolidation in contextual fear conditioning [3], [4], [5] or Morris water Maze tests [6], both of which are hippocampal-dependent. In fear conditioning experiments hippocampal-dependent memory for context was impaired by lipopolysaccharide (LPS) or IL-1β and this effect was prevented using the IL-1 receptor antagonist (IL-1ra) [4].

However, there is also evidence that endogenous IL-1 is actually necessary for memory performance in normal healthy animals. Studies in rats by the Yirmiya group showed that when IL-1ra is administered intracerebroventricularly it impairs performance in the Morris water maze [7]. Using knockout mice, the same group showed that IL-IrKO mice were impaired on both Morris water maze and contextual fear conditioning tasks with respect to wild type control mice [8] and rats have also shown impaired performance in passive avoidance testing when injected with an adenovirus that overexpresses IL-1ra [9]. More recent work also suggests a role for endogenous IL-1 in memory function: deletion of the P2X7 receptor, which is responsible for ATP-dependent IL-1β release, was reported to impair memory in a Y-maze spatial memory task [10]. Taken together all of the above observations suggest that IL-1 is important in learning and memory although its precise functions are still not completely understood. A study that may reconcile these divergent findings on IL-1′s role in memory, demonstrated that IL-1ra disrupted memory performance and that exogenous IL-1β enhanced memory at low concentrations but impaired it at high concentrations [11], suggesting a biphasic dose-response relationship.

Our laboratory has an interest in the role of IL-1 in cognitive function. Since many learning and memory tasks can be confounded by aspects of sickness behaviour, such as decreased locomotor activity, motor speed and consumption of food rewards, our laboratory has developed novel ‘escape from shallow water’ tasks to test working and reference memory in T-maze [12] and Y mazes [13]. Using these and other tests of hippocampal function we examined the possibility that IL-1RI^−/−^ have a generalised hippocampal deficit rather than a specific visuospatial learning deficit. Therefore, we have investigated the nature and degree of cognitive impairment in IL-1RI^−/−^ mice. We also evaluated locomotor activity and anxiety in these mice using open field and elevated plus maze tests. In the light of the behavioural findings we also analysed aspects of hippocampal morphology in IL-1RI^−/−^ and WT animals.

## Materials and Methods

### Animals

In all of the behavioural tasks described below 5–8 month old female or male C57BL/6 and IL-1R1^−/−^ mice were used. C57BL/6 female mice were obtained from Harlan, UK and IL-1R1^−/−^ mice, B6.129S7-Il1r1<tm1Imx>/J (Stock # 003245), were obtained from the inbred colony of Prof. Kingston Mills in the Trinity College Bioresources Unit. In later experiments with male mice, both C57BL6 and IL-1RI^−/−^ mice came from inbred colonies in the same Bioresources unit in TCD. The knockout mice, at the time of their initial arrival from JAX (Bar Harbor, US) had been backcrossed 7 times to a C57 background and thus C57BL/6 mice were an appropriate control strain. All mice were housed in cages of five at 21°C with a 12∶12 hour light dark cycle (lights on from 0800 to 2000) with food and water *ad libitum*. All animal experiments were performed in accordance with Republic of Ireland Department of Health & Children licenses, after ethical approval by the TCD (Trinity College Dublin) Animal Research Ethics Committee. All efforts were made to minimise both the suffering and number of animals used.

### T-maze

To assess hippocampal-dependent working memory we used the paddling T-maze task as previously described [12]. Each mouse was placed in the start arm of the maze with 1 arm blocked such that they were forced to make a left (or right) turn, selected in a pseudorandomised sequence (equal numbers of left and right turns, no more than 2 consecutive runs to the same arm). On taking the turn the mouse could escape from the shallow water and was held in a holding cage for 25 seconds (intra trial interval) during which time the guillotine door was removed and the exit tube was switched to the alternate arm. The mouse was then replaced in the start arm and could choose either arm. The mouse must alternate from its original arm to escape. Correct trials were recorded when the mouse alternated from its original turn and exited the maze. On choosing correctly mice escape from the maze and are returned to their home cage. On choosing incorrectly, the mice were allowed to self-correct to find the correct exit arm. Mice were trained for blocks of ten trials for 12 days (20 minute inter-trial interval).

### Y-maze

To investigate hippocampal dependent reference memory, we used the “paddling” Y-maze visuospatial task as we have previously described [13]. A clear perspex Y-maze mounted on a white plastic base was filled with 2 cm of water at 20 to 22°C, sufficient to motivate mice to leave the maze by paddling to an exit tube at the distal end of one arm, 2 cm above the floor. The mouse exits to a tube in which it is returned to its home cage. Mice were placed in one of two possible start arms in a pseudorandomised sequence for 10 trials and the groups were counter-balanced with respect to the location of the exit and start arm. For any individual mouse the exit arm was fixed. The task was conducted for 20 trials. An arm entry was defined as entry of the whole body, excluding the tail. A correct trial was defined as entry to the exit arm without entering other arms.

### Open Field

To investigate locomotor and rearing activity the open field task was used. Briefly, mice were placed in a box (58 cm×33×19 cm). The number of times the mouse reared and crossed the squares in the box (distance travelled) was recorded for three minutes.

### Elevated Plus Maze

The elevated plus maze task was used to test for anxiety-like behaviour in mice [14]. The maze consists of four arms (two open without walls denoted North/South and two enclosed by high walls denoted East/West) 35 cm long and 5 cm wide. The maze was elevated 45 cm above the surface it was placed on. The mouse was placed in a start arm which was a closed arm. The groups were counterbalanced with respect to start arms. The time spent in the open and closed arms, latency to first emerge from a closed arm and the number of open and closed arm entries were recorded for five minutes. The number of entries and the time spent in the junction were also observed. When at the junction the mouse was regarded as being neither in an open arm nor in a closed arm.

### Morris Water Maze

The water maze consisted of a tank 120 cm in diameter, 60 cm in depth filled with water to a depth of 24 cm. The water was made opaque with the use of a white tempura paint powder (Crafty Devils, UK). A camera was fixed to the ceiling above the water maze and connected to the computer-based tracking programme Ethovision 3.1 (Noldus, Nottingham, UK). Mice were trained using extra maze visual cues to find the location of a hidden platform (15 cm in diameter and 23 cm in height) submerged 1 cm below the water surface and 13 cm from the edge of the pool wall. The pool was divided into four quadrants namely, north, south, east and west; using virtual bisectors using Ethovision. The platform was placed in one of these four different quadrants which were 13 cm from the edge of the pool. The groups were counterbalanced so that there were mice going to each of the four quadrants. The training protocol used was as described by Goshen, consisting of three trials per day per mouse, for 3 days, with a 1 hour break between trials, followed by a probe trial the following day [11]. We then continued training for a further 6 days, followed by a further probe trial. Briefly, the mouse was placed in a holding cage and then gently lowered by the tail into the maze facing the pool wall at different start points. The order of these start points was randomised daily by means of a random sequence generator programme (random.org). The mouse was allowed one minute to find the platform. If, after one minute, the mouse did not locate the hidden platform, the experimenter would direct the mouse to the hidden platform where they would be left for 15 seconds. The mouse was then removed and placed into a heated holding cage and then returned to the home cage for an hour (inter trial interval) until the next trial. The lighting conditions sound and distal visual cues on the walls were controlled and kept constant throughout the experiment. Latency to the hidden platform and distance travelled by the mouse were the parameters recorded. A probe trial was carried out every three days of training to assess memory for the platform location. This consisted of a single one-minute trial in which the hidden platform was removed from the water maze and the % time spent by the mouse in the quadrant where the hidden platform should have been was recorded. On the last day of training, in order to assess visual impairment in the mice, a single flag trial was carried out. A flag was placed onto the hidden platform, in its original position, and the time and distance taken by the mouse to swim to the platform was recorded.

### Spatial Discrimination Beacon Water Maze

The spatial discrimination beacon water maze is an adapation of the water maze task where spatial memory and spatial-discrimination are assessed using two visually identical beacons. [15]. New mice, naïve to behavioural testing, were trained for 8 trials per day, for 3 days, to swim to a black plastic beacon (diameter 15 cm; 24 cm high) sitting on the water surface which had, underneath, a hidden platform for escape from the water (24 trials across 3 days). In this training phase the black plastic beacon+platform (+ve beacon) was moved to different locations in the maze for each trial to ensure that the beacon, rather than other visuospatial cues, was used to locate the hidden platform. A second identical visible beacon, with no hidden platform underneath (−ve beacon), was then introduced to the maze and mice were trained to discriminate between the two identical beacons depending on their allocentric spatial locations. On top of each beacon was a circular (20 cm diameter) piece of laminated white card. Both beacons and the escape platform now remained in the same spatial locations on every trial. The incorrect/decoy beacon was always located in the diametrically opposite quadrant to the correct beacon/platform location. The mice received 8 trials per day for 12 days. A block consisted of 3 days training, i.e 24 trials. First choice accuracy to choose the correct beacon was assessed and the percentage choice of the correct beacon when the start position was furthest from the correct beacon, closest to correct beacon and of equal distance from both beacons were plotted. Total errors were also recorded and analysed (i.e. number of times the mouse swam under the incorrect beacon, as defined by disappearance of the mouse video trace beneath the white circle of the incorrect beacon). A standard probe test was also conducted to assess learning about the spatial location of the platform after 72 trials. Both beacons and the platform were removed from the pool and the mouse allowed to swim freely for 60 sec and % time in the target quadrant plotted.

### Contextual Fear Conditioning

Contextual fear conditioning (CFC) was recorded using a box (40 cm×10 cm×16 cm) with a transparent wall that had a floor containing metal rods which were wired to a shock generator (UGO Basile, Italy). The mice were placed into the box and allowed to explore for 2 minutes. Following exploration, a shock of 0.4 mA was administered for two seconds. An inter trial interval of 2 minutes was followed by a second shock of 0.4 mA for two seconds followed by a further 30 seconds of exploration before returning to the home cage. Fear conditioning was assessed 48 hours later for duration of 5 minutes. Freezing was regarded as the complete absence of movement, except those related to respiration as originally described [16]. Freezing was independently scored by two experimentors, one of whom was blind to the identity of the mice under assesssment.

### Circadian Rhythm

Circadian rhythm was recorded with the use of Phenotyper home cage (45 cm×45 cm) Noldus, Nottingham, U.K) and Ethovision 3.1 (Noldus, Nottingham, U.K). IL-1R1^−/−^ mice and WT mice were left in the Phenotyper home cage for a period of 60 hours (3 nights and 2 days, starting at 8 pm) with free access to food and water. The recordings taken over the 60 hours were then binned into 4 hour periods and presented as 15 consecutive periods, showing night and day activities. Three Phenotyper home cages were used per strain, with five mice in each cage. The total activity for each strain was averaged across these three cages for night and day. Since the software did not allow us to view separate traces for individual animals, statistically the groups are therefore presented as n = 3, but this figure represents the activity of 15 animals of each strain.

### RNA Extraction, Quantitative PCR and ELISA Confirmation of Genotype

A subset of mice were terminally anaesthetised and transcardially perfused with heparinised saline 3 hours after peripheral injection of IL-1β (25 µg/kg) or saline. The hypothalmus and hippocampus of the brains were removed and stored at −80°C until use. As we have previously described [17], total RNA was extracted from the hypothalamus and hippocampus (weighing 25–35 mg) using Qiagen RNeasy® Plus mini kits (Qiagen, Crawley, UK) and the yields were determined by spectrophotometry at 260 nm using a nanodrop (Thermo Scientific, U.S). cDNA was synthesised using a high capacity cDNA reverse transcription kit (Applied Biosystems, Warrington, UK). Two hundred nanograms of total RNA were reverse transcribed in a 10 µl reaction volume. One microliter of the RT reaction (equivalent to 20 ng of RNA) was used for PCR. A standard curve prepared from total RNA from brain treated with LPS was used to allow relative quantification of transcript levels, followed by normalisation to GAPDH expression as we have previously described [18]. To verify that the IL-1R1^−/−^ mice were indeed unresponsive to IL-1β, chemokine (C-X-C motif) ligand 1 (CXCL1) and IL-6 expression were analysed using the following primer pairs CXCL1: forward 5′-CACCCAAACCGAAGTCATAGC-3′ and reverse 5′-AATTTTCTGAACCAA and IL-6: forward 5′TCCAGAAACCGCTATGAAGTTC-3′ and reverse 5′-CACCAGCATCAGTCCCAAGA-3′. Plasma levels of CXCL1 and IL-6 levels were analysed 3 hours post injection of IL-1β (25 µg/kg i.p) or saline using R&D systems duo-set enzyme-linked immunosorbent assays (R&D systems UK) as previously described [19].

### Immunohistochemistry and Quantification

Formalin-fixed, paraffin-embedded sections were cut from the brains of WT (n = 4) and IL-1R1^−/−^ mice (n = 6). Sections were dewaxed in Xylene and Histoclear then rehydrated through a series of decreasing alcohols ranging from 100–70% concentration and treated with 0.2 M boric acid, pH 9, 65°C for 30 min and cooled to room temperature. Non-specific peroxidase activity was eliminated by incubating sections in 1% H_2_O_2_ in ethanol (1 ml H_2_O_2_/100 ml) for 10 minutes. Sections were washed in PBS and blocked using 10% normal horse serum. Sections were incubated with SY38 (Chemicon, CA, USA; 1∶2000), at room temperature overnight. Sections were then washed in PBS before incubation with biotinylated horse anti-mouse secondary IgG (Vector U.K; 1∶200). Sections were then washed in PBS and ABC was applied for 30 minutes followed by diaminobenzidine (DAB) reaction in the presence of ammonium nickel chloride (0.06% w/v) to intensify staining.

The density of synaptophysin staining in IL-1R1^−/−^ mice and WT mice was determined by mono-chrome pixel density analysis of digitally captured images using Cell A imaging software (Olympus, Mason technology, Dublin). Areas of uniform staining within each of the strata of the hippocampal formation (avoiding unstained areas such as blood vessels) were quantified using pixel density analysis. One group of quantifications (comprising all strata) was performed on each section. Transmittance measurements were made at a light intensity within the linear transmittance range (0–255 Lux). In the dorsal hippocampus (bregma −2.0, AP, depth approximately 1 mm) the area of highest transmittance (the corpus callosum) was chosen as an internal standard and all other transmittances were subtracted from this layer. A similar approach was taken in more ventral hippocampus (Bregma −3.1 AP, depth approximately 3 mm), using the CA3 neuronal layer as internal standard to calculating density ratios for radiatum and dentate gyrus polymorphic layer with respect to stratum lacunosum moleculare. The adjusted values for each stratum were then used in ratio calculations to derive a ratio measure of synaptic density as determined by the following equations. For the dorsal ratio: Ratio_DORSAL_ =  (T_corpus callosum_–T_radiatum_)/T_corpus callosum_–T_DG molecular_), while for the ventral region Ratio_VENTRAL_ =  (T_CA3_–T_radiatum OR DG polymorphic_)/T_CA3_–T_lacunosum moleculare_).

Ventricular volume was estimated Using Image J software (NIH, Bethesda, US), and 10 µm sections from WT and IL-R1^−/−^ brains, the ventricles on each section were outlined and an area measurement in pixels obtained. Using a stage micrometer of 1 mm in length the measurement in pixels was converted to mm^2^ (Image J area (pixels)/27.8 = area (mm^2^)). Areas were calculated for sections from each animal at 2.0 and 2.3 mm posterior to Bregma and summed and multiplied by the summed section thickness (2×10 µm: 0.02 mm).

### Statistical Analysis

All statistical analyses were performed using GraphPad Prism 5 for Windows. Data from CFC, elevated plus maze, open field, flag and probe trials and synaptic density were analysed using t-tests while Mann-Whitney tests were used to compare non-parametric ventricular volumes. One Way ANOVA was used to compare cytokine/PCR data while repeated measures two-way ANOVA with strain as between subjects factor and trial block as within subjects factor were used for repeated measures analyses of performance across multiple test sessions. Bonferroni post-hoc tests were used to test for differences at specific time points where main effects of strain or interactions of strain and trial were observed.

## Results

### Y Maze Reference Memory

**Figure 1:**
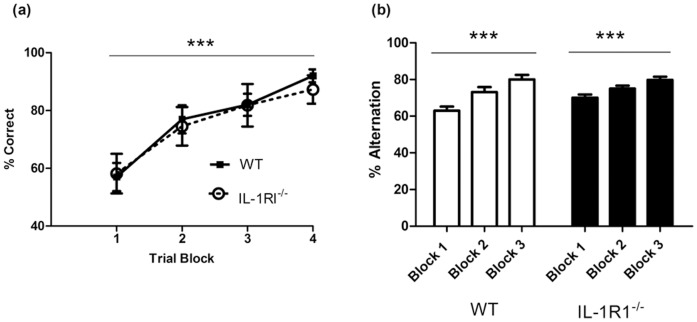
Performance of IL-1R1^−/−^ mice versus WT on a visuo-spatial reference memory task and a working memory task. (a) Visuo-spatial reference memory was assessed using the Y-maze across 20 trials (5 trials per trial block) in IL-1R1^−/−^ (n = 11) compared to WT mice (n = 20). (b) Working memory was assessed in 1L-1R1^−/−^ (n = 11) and WT (n = 15) by T-maze alternation, over 12 days, 10 trials per day with block 1 representing performance of mice on days 1–4, block 2 the performance of mice on days 5–8 and block 3 the performance of mice on days 9–12. Data are shown as mean±SEM and were analysed by two-way repeated-measures ANOVA with strain as between subjects factor and trial block as within subjects factor. Main effects of trial block for both mazes are depicted by ***p<0.001.

Initially all experiments were carried out with female mice. We assessed the performance of IL-1R1^−/−^ mice (n = 11) and WT (n = 20) mice in a visuo-spatial reference memory Y-maze task (fig. 1a) across four blocks of 5 trials. Two-way repeated measures ANOVA with strain as a between subjects factor and trial block as a within subjects factor indicated that there is no impairment on learning of the task in the IL-1R1^−/−^ mice compared to their WT controls. ANOVA revealed no effect of strain (F = 0.10, df 1,29, p = 0.7498), nor an interaction (F = 0.17, df 3,87, p = 0.9191), but there was a significant effect of trial block (F = 18.33, df 3,87, p<0.0001) where performance of both strains improved as training continued.

### T Maze Working Memory

In the T-maze working memory task, a new cohort of animals (n = 15 WT and 11 IL-1RI^−/−^) were trained, for 12 days, to alternate their arm choices to escape from the shallow water maze. The performance of each mouse was assessed for 10 trials per day, for 12 days and these data were then divided into 3 blocks of 4 days (training block 1 (days 1–4) and block 2 (days 4–8) block 3 (days 9–12)). There was no impairment on this task in the IL-1R1^−/−^ mice compared to their controls (fig. 1b). Two-way ANOVA with strain and trial block as factors revealed no significant effect of strain (F = 2.26, df 1,24, p = 0.1280) but a significant effect of trial block (F = 13.24, df 2,72, p<0.0001) and no significant interaction of these 2 factors (F = 1.71, df 2,72, p = 0.2798).

### Contextual Fear Conditioning

**Figure 2:**
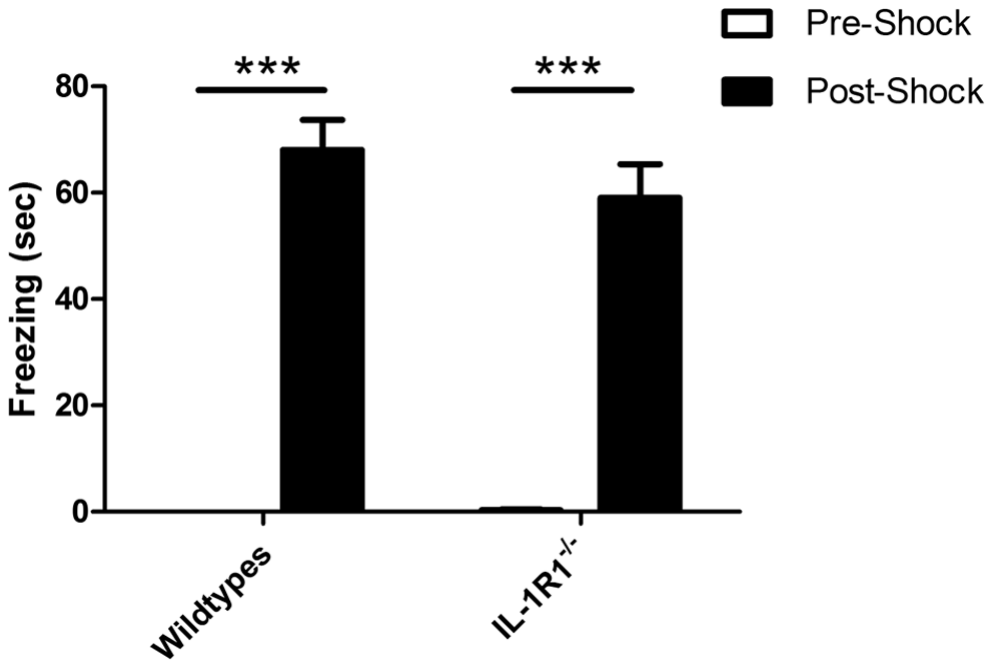
Performance of IL-1R1^−/−^ mice versus WT in the contextual fear conditioning paradigm. The time spent freezing measured across 2 minutes (pre-shock) and 5 minutes, 48 hours later (post shock), following foot shock at 0.4 mA for 2 seconds for IL-1R1^−/−^ mice (n = 10) and WT mice (n = 10) in the fear conditioning paradigm. Data are expressed as mean±SEM and were analysed by t-test, bars representing pre-shock freezing are insufficiently large to be clearly visible. Bonferroni post-hoc tests after a significant effect of treatment in ANOVA analysis showed that both strains showed significant differences from their pre-shock freezing (***p<0.001).

In the contextual fear conditioning paradigm we recorded, for a period of 5 minutes, the time spent freezing 48 hours following exploration and foot shock at 0.4 mA for IL-1R1^−/−^ mice (n = 10) and WT mice (n = 10). The data (figure 2) reveal that the IL-1R1^−/−^ mice show equivalent contextual fear memory compared to relevant controls i.e they spend similar time freezing compared to the WT, indicating a similar recall of the memory of the context in which they received the footshock. Data were analysed by two-way ANOVA with strain and session (pre-shock exploration, post-shock) as factors. There was no effect of strain (F = 1.07, df 1,18 p = 0.3155) and no interaction of session and strain (F = 1.19, df 1,18 p = 0.2889) but there was a significant effect of session (F = 220.03, df 1,18 p<0.0001) indicating that all animals had a memory for the context in which shock was received. Bonferroni post-hoc tests showed that both strains significantly increased freezing between pre-shock and post-shock sessions (p<0.001).

### Increased Open Field Activity and a Decrease in Anxiety like Behaviour in IL-1R1^−/−^ Mice

**Figure 3:**
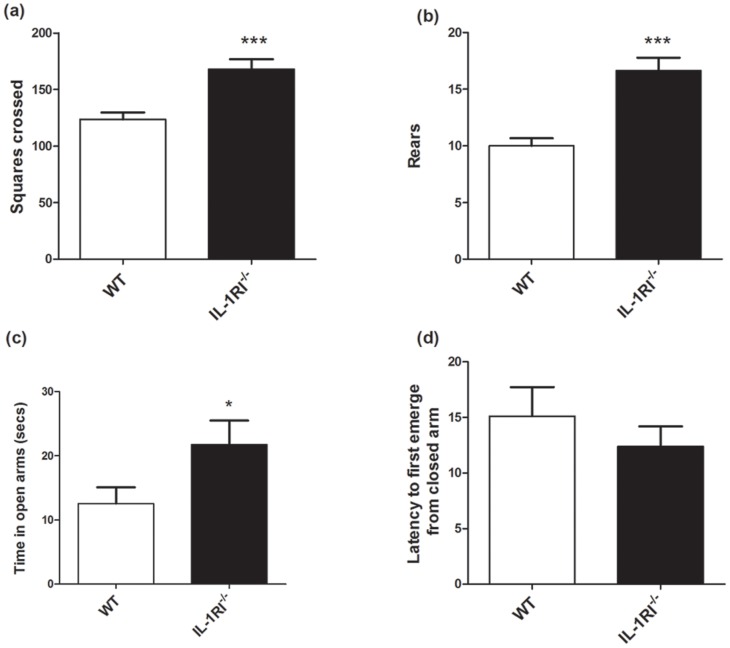
Open field and elevated plus maze activity in IL-1R1^−/−^ and WT mice. (a) Distance travelled in open field in IL-1R1^−/−^ mice (n = 11) compared to WT (n = 19) and (b) the number of rears recorded in open field, across 3 minutes. (c) The time spent in open arms of elevated plus maze and (d) the latency to first emerge from closed arm. Data are expressed as mean ± SEM and significant differences by t -test are denoted by ***p<0.0001 and *p<0.05.

Distance travelled and number of rears in the open field for a period of 3 minutes was recorded. All data in figure 3 are expressed as mean±SEM and significant differences were analysed by unpaired t-tests. The results shown in fig. 3a reveal that IL-1R1^−/−^ mice were more active in the open field compared to controls, showing a significantly greater total distance travelled (p = 0.0002). Their rearing activity in the open field was also significantly higher (p<0.0001, student’s t-test) indicating that exploratory activity is also increased in IL-1RI^−/−^ mice (fig. 3b).

In fig. 3c, in which we investigated the time spent in open and closed arms of the elevated plus maze, the IL-1R1^−/−^ mice spent more time in the open arms of the maze than the WT mice. Students’ t-test confirmed that IL-1R1^−/−^ mice spend significantly more time in the open arms (p<0.05, and significantly less time in the closed arms (data not shown, p<0.05). These data suggest a less anxious phenotype in the IL-1R1^−/−^ mice. Latency to emerge from the initial closed arm was not significantly different between IL-1R1^−/−^ and WT controls (p = 0.467; fig. 3d).

### Learning and Memory in the Water Maze

**Figure 4:**
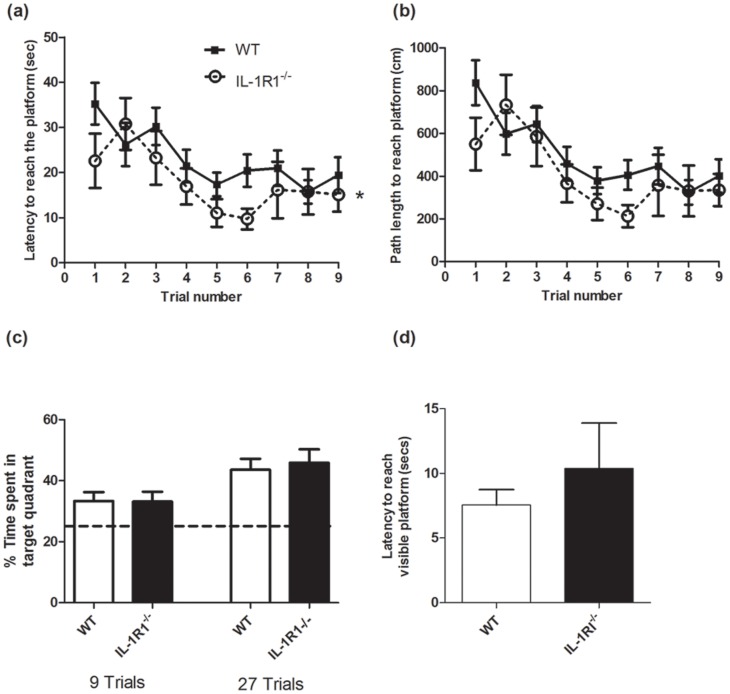
Spatial and non-spatial memory in the Morris water maze in IL-1R1^−/−^ and WT mice. (a) The latency to reach the hidden platform, and (b) the path length were assessed in IL-1R1^−/−^ mice (n = 11) and WT mice (n = 20) in the spatial memory water maze task across 9 trials with 1 hour inter trial interval. (c) Probe trials performed after 9 trials and 27 trials (dotted line shows 25%), and (d) non-spatial memory (flag trial) in IL-1R1^−/−^ mice and WT. Data are expressed as mean±SEM and were analysed by two-way repeated-measures ANOVA with strain as between subjects factor and time as within subjects factor (a, b) or by t-test (c, d). * denotes main effect of strain (p = 0.02).

We assessed the ability of IL-1R1^−/−^(n = 11) and WT mice (n = 20) to locate the hidden platform in the Morris water maze spatial memory task during 3 days of 3 trials with an inter-trial interval of 1 hour (total of 9 trials). The protocol used to test this was that of Avital and colleagues, in order that we might replicate their original findings with the IL-1rKO mouse [8]. The latency (fig. 4a) and path length (Fig. 4b) to reach the platform were recorded. Data are expressed as mean±SEM and were analysed using two-way repeated-measures ANOVA with strain as between subjects factor and trial number as within subjects factor. IL-1R1^−/−^ mice displayed a similar rate of learning in the water maze compared to their controls. The IL-1R1^−/−^ mice showed slightly, but statistically significantly, shorter latencies than the WT to get to the platform. Two-way ANOVA of the latency showed a significant effect of strain (F = 5.93, df 1,29, p = 0.0213) and a significant effect of trial number (F = 3.53, df 8,232, p = 0.0007) but no interaction (F = 0.68, df 8,232, p = 0.7132) and there were no significant differences at any individual time point by Bonferroni post-hoc tests. There was also a slight, but non-significant, difference between the strains on distance swam (no effect of strain (F = 3.34, df 1,29, p = 0.0779; no interaction between strain and trial (F = 0.74, df 8,232, p = 0.6593). Both strains learned the task well (significant main effect of trial number (F = 5.15, df 8,232, p<0.0001). Furthermore, no obvious differences could be observed in the types of routes taken by the 2 strains as assessed by mouse tracking in Ethovision.

In a probe trial conducted after 3 days of training, during which the platform was removed from the pool (Fig. 4c), both strains spent an equivalent time in the target area where the platform was previously located. A further probe trial, conducted after a further 6 days training (Fig. 4d) showed that animals from both strains spend significantly more time in the target area than in any other quadrant (main effect of quadrant F = 29.66, df 2,87, p<0.001). This performance was equivalent in IL-1R1^−/−^ and wild-type animals with p = 0.9635 at 9 trials and p = 0.6970 at 27 trials (unpaired t test of time in target quadrant). In the non-spatial flag trial in which a flag was added to the platform in its existing location, both strains reached the platform more quickly than in the hidden platform version of the maze but there was no significant difference between strains on this parameter (p = 0.3593) as seen in figure 4d. Collectively these data indicate that IL-1RI^−/−^ and WT mice perform with equivalent proficiency in both hidden and visible platform versions of the maze.

### Learning and Memory in the Spatial-discrimination Beacon Water Maze Task

We assessed the ability of IL-1R1^−/−^ mice and their controls to locate the hidden platform in a spatial discrimination beacon water maze task. New mice, naïve to any other behavioural experiment, were used for this task. Briefly mice were trained to discriminate between two identical beacons, one of which had a hidden platform beneath the surface of the water, depending on their allocentric spatial locations. Animals were started from various positions in the maze, some close to the correct beacon, some close to the incorrect beacon and some equidistant from both beacons.

**Figure 5:**
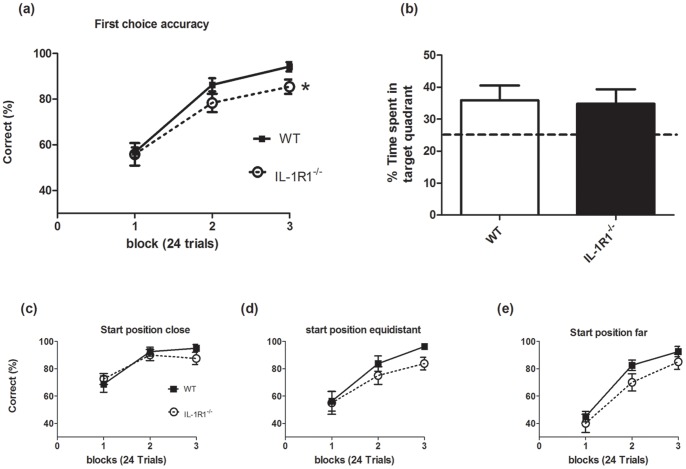
Spatial memory and choice performance on the spatial-discrimination beacon water maze task in IL-1R1^−/−^ and WT mice. IL-1R1^−/−^ and WT mice (n = 10) were trained (8 trials per day for 12 days) to discriminate between two identical beacons (diameter 15 cm; 24 cm high, sitting on the water surface) depending on their spatial locations, one of which had a hidden platform underneath. 1 block = 24 trials. a) First choice accuracy to choose the correct beacon was assessed. b) Probe test performed after 72 trials, with 25% depicted by dotted line. The percentage correct choices on choosing the correct beacon are shown when start position is c) close to correct beacon, d) equal distance from both beacons and e) furthest from correct beacon. * p<0.05 by one-tailed t-test comparison of first choice accuracy across all trials. Data are expressed as mean±SEM and full statistical analysis is described in the text.

Consistent with our previous watermaze experiment, a probe trial conducted after 72 trials (fig. 5b) showed that mice spent more time in the target area than in any other quadrant. ANOVA revealed a main effect of quadrant (F = 13.19, df 2,54 p<0.001). However, this performance was equivalent to wild-type animals with p = 0.8690 (unpaired t-test). Thus, IL-1R1^−/−^ and WT mice learned about the spatial location of the platform to the same extent.

However, given (i) that previous studies have implicated IL-1 in spatial memory performance and (ii) that other aspects of watermaze performance may be more sensitive to disruption of hippocampal function (e.g. impaired synaptic plasticity [15]) than the standard, open field version of the task, we also analysed first choice accuracy for selecting between the beacons and the total errors made. First choice accuracy averaged across all sessions showed a mildly significant difference between strains (t(18) = 1.96; p<0.05, one-tailed). A similar, just significant group difference was also seen for total errors made across all test sessions (t (18) = 1.94; p<0.05, one-tailed). Subsequent ANOVA for first choice accuracy revealed a significant effect of trial block (F = 61.36, df 2,36 p<0.0001), indicating that all animals showed learning across trial blocks (fig. 5a). However there was no significant interaction between trial block and group (F = 0.94, df 2,36, p = 0.4010). Likewise, for total errors, ANOVA demonstrated a significant effect of trial block (F = 68.38, df 2,36 p<0.0001) but no significant interaction between group and trial block (F = 0.63, df 2,36, p = 0.5373). Given that the % choice accuracy results were closer to achieving statistically significant differences, we also performed a 3 way repeated measures ANOVA on these data with strain as a between subjects factor and trial block and start position as within subjects factors. This analysis showed that there was a significant effect of trial block (F = 3.96, df 2,36, p<0.0001), and of start position (F = 9.35, df 2,36, p = 0.005). However, there were no interactions of group and start position, or between group, trial block and start position.

### Systemic and Hypothalamic Cytokine Responses in IL-1R1^−/−^ Mice and WT Mice Challenged with IL-1β

Given the failure to find any substantial spatial learning deficit in the IL-1RI^−/−^ mice, we performed acute IL-1β administration experiments to confirm that the strain was indeed unresponsive to IL-1β. Systemic and CNS cytokine responses in IL-1R1^−/−^ mice and WT mice challenged with IL-1β (25 µg/kg i.p) or saline i.p at 3 hours were assessed by ELISA and RT-PCR. As shown in Table 1, IL-6 and CXCL1 were detectable at very low levels in WT mice treated with saline or in IL-1R1^−/−^ mice treated with IL-1β but these molecules were robustly expressed in WT mice treated with IL-1β. Plasma cytokine responses to IL-1β were equally limited in both male and female IL-1RI^−/−^ mice. Similarly we assessed the CNS transcription of mRNA for these IL-1β -sensitive genes. IL-1β induced robust transcription of both cytokine transcripts in WT animals but did not produce any change from basal levels in either male or female IL-1RI^−/−^ mice. The same patterns were true for hippocampal transcription (data not shown). Thus, IL-1RI^−/−^ mice, of both genders, are completely unresponsive to IL-1β stimulation. We also analysed these animals for IL-1β protein (plasma) and mRNA (hypothalamus) and found no evidence of elevated basal IL-1β in these animals.

**Table 1:** Cytokine and chemokine transcription and synthesis.

	WT+Saline	WT+IL-1β	IL-1R1−/−+IL-1β	
			female	male
IL-6 Plasma	63±8	2442±240	3±1	<1
CXCL1Plasma	145±14	222,680±13,080	196±22	104±31
IL-6 mRNA	<0.0015	0.113±0.015	<0.0015	<0.0015
CXCL1mRNA	<0.003	0.252±0.0328	<0.003	<0.002

Systemic and hypothalamic cytokine responses in IL-1R1^−/−^ mice and WT mice challenged with IL-1β. Plasma was prepared from whole blood of WT and IL1R1^−/−^ mice 3 hours post treatment with IL-1β (25 µg/kg i.p.) or saline and analysed by ELISA. CNS cytokine transcription was assessed by quantitative PCR on cDNA synthesised from total RNA isolated from hypothalamic tissue at the same time. All data are expressed as mean±SEM and were analysed by one way ANOVA where n = 5 for WT/saline, n = 4 for WT/IL-1β and n = 6 for female IL-1R1^−/−^ mice and n = 5 for male IL-1R1^−/−^ mice challenged with IL-1β.

### Circadian Rhythm

**Figure 6:**
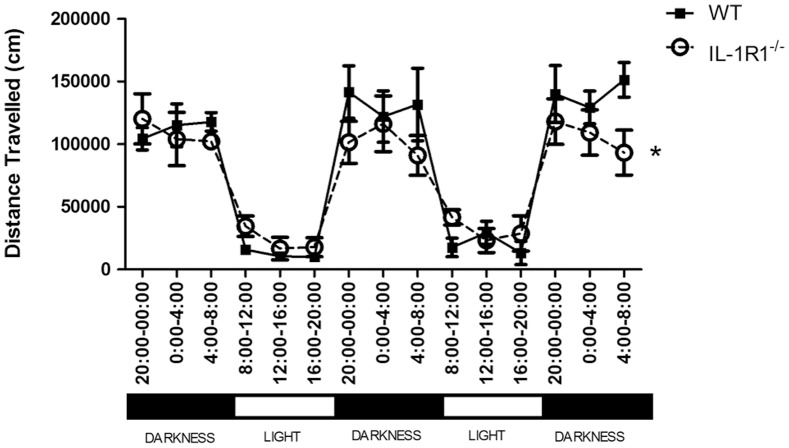
Circadian rhythm of female WT and IL-1R1^−/−^ mice. The activity of female WT (n = 15) and female IL-1R1^−/−^ (n = 15) mice was recorded using the Phenotyper home cage over a period of three nights and two days. Data are plotted in 4 hour blocks and are expressed as mean±SEM and were analysed by two way repeated measures ANOVA with strain as between subjects factor and time as within subjects factor. There was no effect of strain but a significant time × strain interaction, which is denoted by *p<0.0003.

In some previous published studies in which cognitive changes have been observed in IL-1RI^−/−^ mice, animals were housed under a reversed light cycle such that housing rooms are dark during the daytime and cognitive experiments were therefore performed during the animals’ ‘subjective night’ or more active period. We reasoned that it may be informative to assess the circadian rhythm of IL-1R1^−/−^ mice compared to WT controls. We measured total activity of a cohort of 15 female IL-1R1^−/−^ mice and 15 female WT in PhenoTyper home cage, 45 cm×45 cm, (Noldus, UK) captured by Ethovision (for statistical purposes these constitute n = 3 per group because each phenotyper cage monitored total activity for 5 animals each). As expected, over a period of three nights and two days plotted in 4 hour blocks, both the IL-1R1^−/−^ and WT mice were clearly more active in the night phase compared to the day (fig. 6) and there was a main effect of time (F = 57.09, df 14,56, p<0.0001). Although distance travelled was not affected by strain (F = 0.27, df 1,4, p = 0.6319) there was a significant interaction of time and strain (F = 3.55, df 14,56, p = 0.0003). This may reflect that IL-1RI^−/−^ mice travelled shorter distances in the dark phase than did WT mice, although there were no significant differences of individual time points by Bonferroni post-hoc comparisons.

### Effect of Gender

**Figure 7:**
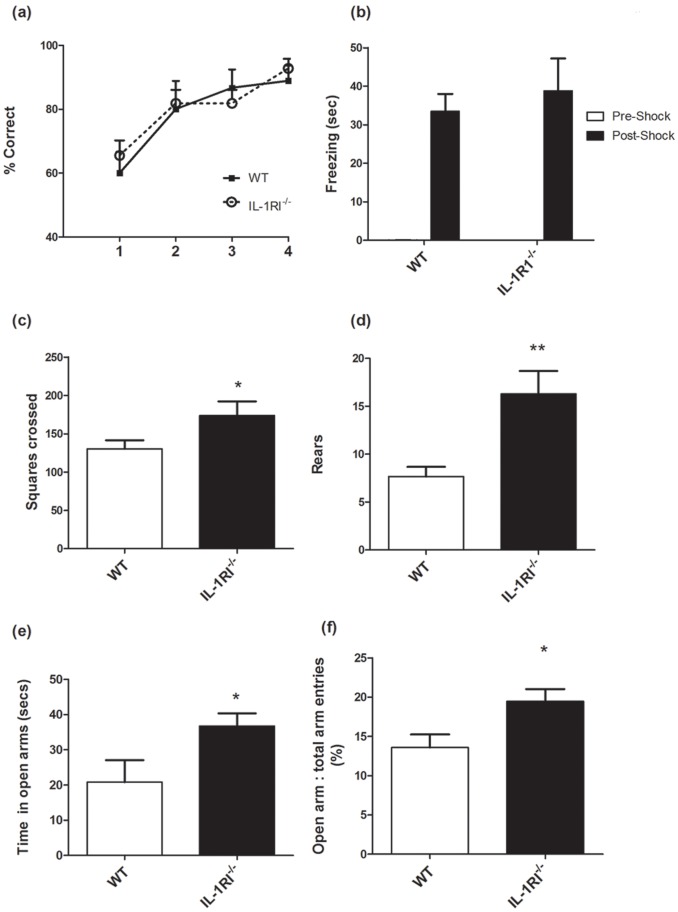
Performance of male IL-1R1^−/−^ mice versus male WT on a battery of tasks. (a) Visuo-spatial reference memory was assessed using the Y-maze across 20 trials (n = 5 per trial block) in male IL-1R1^−/−^ (n = 11) compared to male WT mice (n = 9). (b) The time spent freezing before (too small to be clearly visible), and 48 hours following, 0.4 mA foot shock for male IL-1R1^−/−^ mice and WT mice in the fear conditioning paradigm. (c) The number of squares crossed in 3 minutes in the open field and (d) the number of rears recorded in open field, across 3 minutes (e) the time spent in open arms of the elevated plus maze and (f) entries into open arms as a proportion of total arm entries (for male and females combined). Data are expressed as mean ± SEM and figure (a, b) were analysed by two way repeated-measures ANOVA with strain as between subjects factor and time/block as within subjects factor and all other figures were analysed by one-tailed t-test and significant differences are denoted by ** p<0.001 and *p<0.05.

Since previous published studies on cognitive deficits in IL-1RI^−/−^ mice were carried out in males, it was important to verify that our results with female mice generalised to males. The Y-maze, open field, fear conditioning and elevated plus maze were thus repeated in male IL-1R1^−/−^ and male WT mice. We assessed the performance of male IL-1R1^−/−^ mice (n = 11) and male WT (n = 9) mice on a visuo-spatial reference memory Y maze task (fig. 7a) across four blocks of 5 trials. Two-way repeated measures ANOVA with strain as between subjects factor and trial block as within subjects factor showed that there was no impairment on learning of the task in male IL-1R1^−/−^ mice compared to their controls (WT). There was no effect of strain (F = 0.16, df 1,18, p = 0.6935) or any interaction (F = 0.37, df 3,54, p = 0.7759) but a significant effect of trial block (F = 10.40, df 3,54, p<0.0001) where performance of both strains improved as training continued.

We recorded, for 5 minutes, the time spent freezing 48 hours following exploration and foot shock at 0.4 mA in the fear conditioning paradigm. These data reveal that the male IL-1R1^−/−^ mice show equivalent, unimpaired, contextual fear memory compared to their controls i.e they spend similar time freezing compared to the WT (figure 7b). Data were analysed by two-way ANOVA with conditioning (pre vs. post-shock levels) and strain as factors. There was no effect of strain (F = 0.26, df 1,18 p = 0.6151) and no interaction between conditioning and strain (F = 0.27, df 1,35 p = 0.6092) but there was a significant effect of conditioning (F = 47.99, df 1,35 p<0.0001).

Distance travelled and the number of rears in the open field for a period of 3 minutes was recorded. Data are expressed as mean±SEM and significant differences were analysed by unpaired t-tests. Based upon an *a priori* prediction of increased activity in IL-1R1^−/−^ mice, arising from our results in females, we compared open field activity (fig. 7c) in the male IL-1R1^−/−^ versus WT mice by one-tailed t-test and demonstrated a significantly increased activity (p = 0.0362). Similarly, as shown in figure 7d, there was a significant increase in rearing activity in the IL-1R1^−/−^ mice (p = 0.0035). Figure 7e shows that the IL-1R1^−/−^ mice spend a significantly longer time in the open arms of the elevated plus maze compared to the WT mice (p<0.05). This increased time in the open arms suggests that male IL-1R1^−/−^ mice are less anxious, as was observed in the female mice (fig 3c). Since increased time in open arms might be a product of more arm entries in a general sense (ie since they show hyperactivity) we addressed this possibility by examining, in mice of both genders combined, the number of open arm entries as a ratio to the total arm entries. (fig. 7f). These data show that a greater proportion of the total arm entries of the IL-1R1^−/−^ mice are into open arms than those of WT mice (p = 0.0160 t-test), thus indicating a decrease in anxious behaviour in the IL-1RI^−/−^ mice.

### Ventricular Volume and Synaptic Density

**Figure 8:**
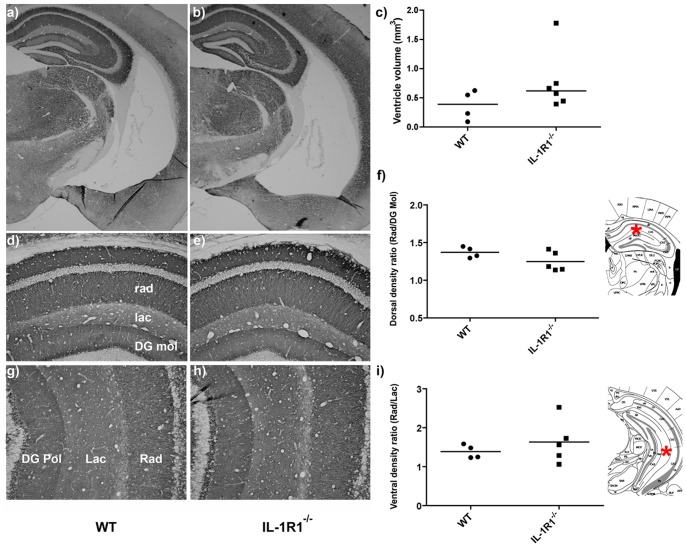
Ventricular volume and synaptic density in WT and IL-1R1^−/−^ mice. (a–c) Representative images of the hippocampus and lateral ventricle of WT (a) and IL-1R1^−/−^ (b) brain. Ventricular volume in WT (n = 4) and IL-1R1^−/−^ mice (n = 6) was assessed using Image J Software (c). Areas were calculated for sections from each animal at 2.0 and 2.3 mm posterior to Bregma and summed and multiplied by the summed section thickness (2×10 µm: 0.02 mm). Data are non-parametric and have been presented as dot plots with median value denoted by the bar. (d–i) Synaptophysin labelling in the dorsal (see inset *) and more ventral hippocampus (approximately −3.0 mm from surface of brain; see inset *) of WT (d, g) and IL-1R1^−/−^ mice (e, h). Synaptic density analysis of the dorsal hippocampus: ratio of stratum radiatum to dentate gyrus molecular (f) and ventral hippocampus ratio of stratum radiatum to stratum lacunosum moleculare (i) for WT (n = 4) and IL-1R1^−/−^ (n = 5).

The ventricular volume in IL-1R1^−/−^(n = 6) and WT mice (n = 4) was assessed with the use of ImageJ. Figure 8c, shows that there is no significant difference in the ventricular volume between WT or IL-1R1^−/−^ mice (p = 0.1714) as assessed by MannWhitney test following Kolmogorov–Smirnov test, although there were was a trend towards enlarged ventricular volume in the IL-1RI^−/−^.

We also investigated whether there was a difference between the two strains in synaptic density in regions of the dorsal and ventral hippocampus. In figures 8f and 8i where mean values density ratios (layer of interest as ratio to reference layer) are presented, there were no differences in synaptic density in the stratum radiatum between the IL-1R1^−/−^ mice and WT mice in either dorsal hippocampus (Bregma −2.0 mm AP, 1.2 mm DV) or in more ventral hippocampus (Bregma −3.1 mm AP, −3mm DV from surface of brain). Similar results were observed for the DG polymorphic layer (data not shown). Unpaired t tests showed that there were no statistically significant differences (p≥0.1324), although there was generally more variability in values in the IL-1RI ^−/−^ animals.

## Discussion

In the current study we were unable to detect significant differences between IL-1R1^−/−^ and WT mice on multiple hippocampal-dependent, spatial memory tests including T-maze working memory, Y and Morris water maze tests of reference memory, and contextual fear conditioning. There was, however, a subtle deficit in choice behaviour in IL-1R1^−/−^ mice on a spatial discrimination beacon test in the watermaze. IL-1R1^−/−^ mice also showed a significantly increased distance travelled and rears in a novel open field and showed decreased anxiety in the elevated plus maze. These effects were similar in male and female mice.

### IL-1 in Anxiety and Novel Environment Hyperactivity

The present study demonstrates that IL-1RI^−/−^ mice show increased locomotor activity with respect to wild-type controls in open field experiments. However the activity data in the continuous activity monitoring boxes suggest that their general activity is not significantly different to WT controls, indicating that this increased activity is likely to reflect a response to novelty rather than increased activity per se. Consistent with this, rearing activity, which is an exploratory behaviour, was also significantly increased in the open field. This type of increased activity in open field could potentially indicate a spatial learning impairment in these mice, although our data from the spatial memory tests performed do not support this possibility. Furthermore, our data from the elevated plus-maze test suggest that IL-1RI^−/−^ are actually less anxious than wild type controls: knockout animals spent significantly more time in the open arms and less time in the closed arms than WT controls. These data are indicative of decreased anxiety in these mice. Although the time spent in open arms could potentially be confounded by the increased locomotor activity already described, we also found that when corrected for total activity (i.e total arms entered) the arm entries into open arms as a proportion of total arm entries maintained the stronger tendency to visit the open arms in IL-IRI^−/−^ mice (figure 7f).

Since general hyperactivity does not appear to be the explanation of the current data, decreased anxiety in IL-1RI^−/−^ mice appears the more likely account. The finding that the disruption of IL-1RI expression is associated with a decrease in anxiety is consistent with one previous study in IL-1RI^−/−^ mice [20]. Since it is well described that acutely elevated IL-1β induces anxiety [21], [22] and that the anhedonic and decreased neurogenesis effects of chronic unpredictable stress are mediated by IL-1 [23], it seems reasonable to argue for a role for endogenous IL-1 in inducing anxiety in normal animals and that decreased responsiveness to endogenous IL-1 drives decreased anxiety. Since increased activity in a novel open field likely reflects the triumph of exploratory drive over anxiety in an unfamiliar environment, it might be that the increased activity in the open field and the increased time in the open arms of the elevated plus maze are both expressions of decreased anxiety and that this is brought about by decreased endogenous IL-1 action in brain anxiogenic centres. It is, of course, impossible to pinpoint the locus of reduced anxiety in the brain of a global knockout animal. However, there is considerable evidence to suggest an important role for the hippocampus, and particularly the ventral subregion of the hippocampus, during ethological, unconditioned tests of anxiety like the elevated plus maze and the open field [24], [25], [26], [27], [28], [29], [30]. It has been argued that during these unconditioned tests, anxiety is generated by the approach/avoidance conflict experienced by the animals [27], [31]. For example, on the elevated plus maze the mouse is faced with the choice of either approaching and exploring the open arms, or avoiding them and staying safe in the enclosed sections of the maze. An important component of the anxiety response in normal animals is the behavioural inhibition of motor activity and, in particular, inhibition of the approach response towards the open arms [31], [32]. In the present study IL-1RI^−/−^ mice were less able to inhibit the approach response during these approach/avoidance conflict tests. It is notable that the IL-1RI^−/−^ mice were also less able to inhibit their approach responses to the decoy beacon during the spatial discrimination version of the MWM task. Although we should be cautious about over-interpreting what is only a mildly significant effect in the beacon watermaze task, it nevertheless may help to identify a key psychological process (behavioural inhibition of approach responses) which could be important, not only in interpreting certain tests of cognition but also during anxiety, and which could also be a key target for modulation in sickness behaviour.

Preliminary investigations into a neuropathological basis for these decreased anxiety effects in IL-1RI^−/−^ mice were not definitive, with no signficant effects on ventricular volume or hippocampal synaptic density in dorsal or more ventral regions, although changes in the lamination of the hippocampus in ventral regions make the analysis conducted here difficult in the most ventral regions. There is a recognised double dissociation between the dorsal hippocampus and ventral hippocampus with respect to spatial memory and anxiety [24] and it will be worth investigating, in IL-1RI^−/−^ and littermate controls, the possibility that there are more pronounced structural or morphological differences in the anxiety-associated ventral hippocampus.

It is also important to note that the described differences in open field activity and decreased anxiety also represent important potential confounding factors in studies of cognitive function. That is to say, in cognitive tasks that are aversively motivated, such as the MWM and contextual fear conditioning, an animal with decreased anxiety may show differences in these cognitive tasks because of its different response to the aversive stimulus rather than because of some cognitive impairment. Aspects of sickness behaviour can significantly confound cognitive testing [33] and given the divergent findings on endogenous IL-1 in cognitive function in this and previously published studies, altered levels of anxiety and activity may be a key consideration here. Other important considerations are discussed below.

### Endogenous IL-1 in Learning and Memory

It has been reported that endogenous IL-1 plays an important role in hippocampal-dependent learning and memory. Avital and colleagues showed that IL-1rKO mice are impaired on the Morris water maze task, displaying a slower rate of learning (longer latencies to reach hidden platform in this spatial memory paradigm) compared to the WT [8]. The authors also showed that the IL-1rKO mice were impaired on contextual fear conditioning, displaying significantly less freezing than their WT controls 48 hours post-conditioning, although these animals were not impaired on an auditory-cued conditioning task. Subsequent studies by the same group showed that transgenic mice engineered to express IL-1ra also showed impaired learning in both the MWM and CFC tasks [11]. There are somewhat similar studies in rats showing that IL-1ra expressed by replication-deficient adenovirus, also impairs learning in a passive avoidance task [9]. However, in the present study we have found little evidence for a role of IL-1RI in hippocampal-dependent spatial learning in normal animals.

The current experiments began by demonstrating normal, spatial working memory performance in a T-maze escape from water alternation task in the IL-1RI^−/−^ mice. Previous studies have suggested that spatial working memory and spatial reference memory reflect distinct psychological processes, subserved by different neural mechanisms. Therefore, the lack of effect on alternation behaviour in our mice compared with the deficits reported by Avital and colleagues could reflect the different nature of the memory processes involved. However, a simple visuospatial Y-maze spatial reference memory task, which like the MWM, relies on use of visuospatial cues to escape water, was also solved equally easily by WT and IL-1RI^−/−^ mice. We next performed contextual fear conditioning experiments but we found robust and equivalent freezing responses in WT and IL-1RI^−/−^ in male and female mice.

In an attempt to replicate previous studies we also assessed the Morris watermaze (MWM), following the exact protocol of Avital et al [8] (3 trials per day for 3 days with a 1hr inter trial interval, as compared to the five day training period more often used). Avital and colleagues [8] show relatively rapid learning of the task by WT animals, already showing quite stable performance by trial 4, and slower learning in the IL-1R1^−/−^ mice: showing longer latencies to the hidden platform and travelling greater distances to find it. We did not observe such rapid learning of the task in WTs in our study and the probe test performed after 9 trials of spatial training revealed only a slight preference for the training/goal quadrant (33% in target quadrant). This rapid learning in controls, rather than ‘slow’ learning by the IL-1R1^−/−^ may constitute a key difference between our studies and those of Avital and colleagues. Given this rather short training protocol of 3 trials per day for 3 days with a 1hr inter trial interval and the concomitant mild preference for the goal quadrant displayed by both groups, we continued training after the 9 trials for a further 18 trials. Even after this much longer training session we found that the IL-1R1^−/−^ mice were not significantly different to WTs on this spatial reference memory task. Importantly, after this extended period of training, both groups now showed a much stronger preference for the training quadrant in the probe test (>40% time in training quadrant). Indeed, IL-1RI^−/−^ and WT animals performed similarly on probe trials performed after 9 trials and 27 trials. They were also similar on the visible ‘flag’ trial. Therefore, it is clear that IL-1RI animals can learn this task and in the current study do so at the same rate as WT controls.

In a recent study by Bannerman and colleagues [15] it was shown that performance on a spatial discrimination version of the MWM task is more sensitive to disruption of hippocampal synaptic plasticity than performance on the standard open field version of the MWM. Mice lacking NMDARs in the dentate gyrus and CA1 subfields of the hippocampus were capable of learning the spatial location of the platform but were less accurate at discriminating between two visually identical beacons, one of which signified the position of the escape platform as defined by the allocentric, extramaze spatial cues. Given the deficits in hippocampal synaptic plasticity reported in the IL-1R1^−/−^ mice [8] we therefore assessed these animals on the spatial discrimination/beacon version of the watermaze task. During standard probe tests in which both beacons and the escape platform were removed from the pool, both WT and IL-1R1^−/−^ mice again showed an equivalent preference for the training quadrant, suggesting that both groups had learned about the spatial location of the platform to the same extent. In contrast, the IL-1R1^−/−^ mice displayed a subtle increase in the number of times they approached the wrong (decoy) beacon (both in terms of first choice accuracy and total errors). Bannerman et al., [15] argued that the impairment in spatial discrimination performance in mice lacking hippocampal NMDARs was not due to an impairment in associative spatial learning but instead reflected an inability to behaviourally inhibit the very strong conditioned response that the mice have to swim to the first beacon that they encounter. Given the normal acquisition of the standard, open field version of the MWM task in the IL-1R1^−/−^ mice and their ability to learn about the spatial location of the platform during the beacon task, their deficit in choice behaviour may also reflect impaired behavioural inhibition rather than impaired associative spatial learning, as discussed above.

### Basis for Divergent Effects in IL-1RI^−/−^ Studies

The present results are apparently at odds with those studies showing that IL-1rKO mice are impaired in visuospatial memory in both the standard, open field MWM and during CFC [8]. There were several explanations that might have accounted for these divergent data. Our first thought was that since our study was initially performed in females, and failed to replicate cognitive deficits reported in males, this might signify an interesting interaction with gender. However, further experiments conducted in males in the Y-maze and in contextual fear conditioning confirmed that the cognitive performance was equivalent in knockouts and wild types on these two hippocampal-dependent tasks. Furthermore, the differences between IL-1RI^−/−^ and wild types observed in open field and elevated plus maze experiments were the same in males as they were in females. These data indicate that gender is not an important factor in either the cognitive or anxiety tests used in the current study.

It is also of note that Goshen and co-workers demonstrated the effect of IL-1β pathways on behaviour using mice raised in reverse cycle [11]. Thus, their experiments were conducted during the mice’s more active period. Contrary to Goshen et al. the animals we used for our study were not raised in reverse cycle, and we thus performed all our behavioural studies during their less active period. Interestingly, several studies have demonstrated the influence of sleep and time of day on hippocampal-dependent plasticity and subsequent behavioural abilities [34], [35], [36], [37]. In addition, a recent paper revealed that, under physiological conditions, time of day influenced IL-1β and IL-1RI expression and speculated that IL-1 may contribute to the basal functioning of the suprachiasmatic nucleus (SNC) clock [38]. The SCN is known to strongly modulate hippocampal-dependent plasticity [39]. Thus, one might hypothesize that IL-1β may have a role in influencing the daily SNC-controlled oscillation of hippocampal-dependent memory performance. We did perform some experiments to assess the circadian rhythm of locomotor activity in IL-1RI^−/−^ and wild type animals and these studies showed that overall distance travelled was equivalent in both strains and that both strains showed the expected dark phase peak in activity. However, the interaction of strain and time did indicate that IL-1RI^−/−^ were perhaps slightly less active in the dark phase and perhaps slightly more active in the light phase. This leaves open the possibility that the strains may show other behavioural differences if tested during subjective night but does not offer an obvious explanation for the differences in cognitive function observed in our studies with respect to those of the Yirmiya group.

Another possibility that might explain differences between findings at different sites is the health status of the animals. IL-1 is a key mediator in immune responses and IL-1RI^−/−^ animals are more prone to certain infections [40]. Therefore it is plausible that colonies may carry low-grade infections and that these may vary from one animal unit to another. In the current study no animal displayed any sign of sickness behaviour, but it remains possible that infection in animal colonies might suppress activity in wild-type animals and that this effect might be different in IL-1R1^−/−^ mice. Undetected infection in the colony is an additional factor that should be considered to interrogate differences between divergent results in different animal units.

### Background Strain Differences

Another possible reason for the divergent results observed between our study and those of the Yirmiya group is a difference in strains used in these studies. In our studies female or male Interleukin 1 receptor 1 knock-out mice (IL-1R1^−/−^; B6.129S7-Il1r1tm1Imx/J; Stock number 003245) were obtained from an in-house colony, originally imported from Jackson Laboratories, USA. The initial generation of these mice [41] involved both 129Sv and C57 strains. Briefly, a null mutation in *Il1r1* was generated by homologous recombination in 129/SvEv AB1 ES cells and targeted mutant mice were subsequently backcrossed 7 times to C57BL/6 background (that was not C57BL6/J). The IL-1RI^−/−^ animals supplied by JAX have remained at generation N5+N2F2 (since October 2009). As such they were initially a C57/129S7 hybrid and were backcrossed 5 times onto a C57 background before importation by JAX and a further 2 times onto a C57BL6/J background after importation by JAX. We have subsequently maintained them as an inbred colony and have used C57BL6 as our control strain. The studies of Koo and Duman have also been performed with these mice. A recent (March 2011) 32 single nucleotide polymorphism panel analysis of the JAX mice, with 27 markers covering all 19 chromosomes and the X chromosome, and a further 5 markers distinguishing between C57BL/6J and C57BL/6N sub-strains, was performed on the re-derived living colony at The Jackson Laboratory Repository. 26 of 27 markers throughout the genome indicate a C57BL/6 genetic background (http://jaxmice.jax.org/strain/003245.html). Furthermore, 3 of 5 markers that distinguish C57BL/6J from C57BL/6N indicated that the mice originally sent to JAX were on a C57BL/6N genetic background or a mixed C57BL/6J;C57BL/6N genetic background. As such, we would argue that C57BL/6 mice are an appropriate control strain. It is of interest that the studies of Koo and Duman were also performed with C57BL/6 controls, but not C57BL6/J originating from JAX. Our data demonstrating decreased anxiety, increased activity and a limited effect on cognitive performance are consistent with the Koo and Duman studies [20] and divergent from those of the Yirmiya group, which were performed with 129×C57 crosses. The Yirmiya group have used a different IL-1R1^−/−^ animal: the B6;129S1-*Il1r1tm1Roml*/J (Stock Number:003018), which is maintained as a hybrid strain (129S1/Sv * C57BL/6 cross). Thus a 129/Sv X C57BL/6 cross was used as the control strain for their initial and subsequent studies [3], [7], [8], [11]. In simple terms the IL-1RI knockouts and the control animals in those studies are 50/50 129/C57 and thus while all three research groups have used appropriate control strains for their respective IL-1RI^−/−^ mice, the effects of deletion of IL-1RI^−/−^ may be different depending on the background strain of mouse.

There is evidence for strain differences on performance of hippocampal-dependent tasks. Early studies of 129 mice found them to be behaviourally and cognitively deficient with respect to C57 mice [42]. Wolff et al showed better performance of C57 mice than 129 mice on some paradigms in the Morris water maze and worse performance than 129 mice in other paradigms in the same maze [43] Other studies found different latencies to find the hidden platform, but this was explained by more rapid swim speeds in C57 mice and indeed distance travelled revealed equivalent rates of learning in these strains [44]. Interestingly, there is also some evidence that 129×C57 hybrids learn the Morris water maze more rapidly than either 129 or C57 strains [45], which is consistent with the rapid MWM learning observed by [8]. To our knowledge, there are no studies in which these IL-1RI^−/−^ mice have been compared to littermate controls on tasks of learning and memory, and behavioural features such as locomotor activity and anxiety. This is obviously a weakness in this literature, including the current study, and it is necessary to perform such experiments in order to resolve differences between IL-1RI deletion in different backgrounds. The current studies evolved from those in which we noticed intact working memory performance in IL-1RI^−/−^ mice, performed with inbred colonies, and we simply continued the analysis with the existing colonies. It is now clear, in the light of the inconsistencies reported in the literature and our own current data that further studies on the role of endogenous IL-1RI should be performed with littermate controls.

We have been robust in performing and repeating the current experiments in larger numbers of animals than earlier studies, using a larger battery of cognitive tasks than in any previous study with IL-1RI knockout mice, and with mice of both sexes. We believe we can confidently state that the loss of IL-1RI does not affect hippocampal-dependent learning and/or memory in these mice, at least under the conditions under which we ran our experiments.

It is worth pointing out that there are also experiments with IL-1ra [7] and others with P2X7 receptor knockouts [10] that also suggest a role for endogenous IL-1 in learning and memory and the current experiments do not speak to those data. This study was not designed to examine the hypothesis that IL-1 is important in learning and memory, but rather to characterise the IL-1R1^−/−^; B6.129S7-Il1r1tm1Imx/J mouse strain. It is clear that we have not ‘disproved’ the idea that endogenous IL-1 is important in learning and memory, but we believe the current data raise sufficient questions to merit a careful re-evaluation of the cognitive data in IL-1RI^−/−^ mice. It is now important to determine the experimental conditions under which IL-1 is important for learning and memory performance, and conversely the conditions when it is not essential. In performing such experiments it will be important to address the possibility that locomotor hyperactivity and anxiety (or lack of anxiety) are confounding factors that could be misinterpreted as cognitive impairments.

## Conclusion

In conclusion, these results suggest that, contrary to prior published studies, IL-1R1^−/−^ mice are not robustly impaired on hippocampal-dependent learning and memory tasks but do display a lower level of anxiety compared to WT mice. The study suggests that IL-1 signalling is involved in normal anxiety responses and neuroanatomical and neurochemical investigations for the basis of these effects should continue in knockout animals and littermate controls.
